# Paediatric Gliomas: BRAF and Histone H3 as Biomarkers, Therapy and Perspective of Liquid Biopsies

**DOI:** 10.3390/cancers13040607

**Published:** 2021-02-04

**Authors:** Jean Yin Tan, Ipalawattage Vindya Stephnie Wijesinghe, Muhamad Noor Alfarizal Kamarudin, Ishwar Parhar

**Affiliations:** Brain Research Institute Monash Sunway, Jeffrey Cheah School of Medicine and Health Science, Monash University Malaysia, Bandar Sunway 47500, Selangor, Malaysia; jytan65@student.monash.edu (J.Y.T.); vsipa1@student.monash.edu (I.V.S.W.); muhamadnoor.alfarizal@monash.edu (M.N.A.K.)

**Keywords:** paediatric gliomas, *BRAF* mutation, *BRAF* V600E, Histone H3, H3K27M, gliomas, diffuse intrinsic pontine glioma

## Abstract

**Simple Summary:**

Gliomas are major causes of worldwide cancer-associated deaths in children. Generally, paediatric gliomas can be classified into low-grade and high-grade gliomas. They differ significantly from adult gliomas in terms of prevalence, molecular alterations, molecular mechanisms and predominant histological types. The aims of this review article are: (i) to discuss the current updates of biomarkers in paediatric low-grade and high-grade gliomas including their diagnostic and prognostic values, and (ii) to discuss potential targeted therapies in treating paediatric low-grade and high-grade gliomas. Our findings revealed that liquid biopsy is less invasive than tissue biopsy in obtaining the samples for biomarker detections in children. In addition, future clinical trials should consider blood-brain barrier (BBB) penetration of therapeutic drugs in paediatric population.

**Abstract:**

Paediatric gliomas categorised as low- or high-grade vary markedly from their adult counterparts, and denoted as the second most prevalent childhood cancers after leukaemia. As compared to adult gliomas, the studies of diagnostic and prognostic biomarkers, as well as the development of therapy in paediatric gliomas, are still in their infancy. A body of evidence demonstrates that B-Raf Proto-Oncogene or V-Raf Murine Sarcoma Viral Oncogene Homolog B (*BRAF*) and histone H3 mutations are valuable biomarkers for paediatric low-grade gliomas (pLGGs) and high-grade gliomas (pHGGs). Various diagnostic methods involving fluorescence in situ hybridisation, whole-genomic sequencing, PCR, next-generation sequencing and NanoString are currently used for detecting *BRAF* and histone H3 mutations. Additionally, liquid biopsies are gaining popularity as an alternative to tumour materials in detecting these biomarkers, but still, they cannot fully replace solid biopsies due to several limitations. Although histone H3 mutations are reliable prognosis biomarkers in pHGGs, children with these mutations have a dismal prognosis. Conversely, the role of *BRAF* alterations as prognostic biomarkers in pLGGs is still in doubt due to contradictory findings. The *BRAF* V600E mutation is seen in the majority of pLGGs (as seen in pleomorphic xanthoastrocytoma and gangliomas). By contrast, the H3K27M mutation is found in the majority of paediatric diffuse intrinsic pontine glioma and other midline gliomas in pHGGs. pLGG patients with a *BRAF* V600E mutation often have a lower progression-free survival rate in comparison to wild-type pLGGs when treated with conventional therapies. BRAF inhibitors (Dabrafenib and Vemurafenib), however, show higher overall survival and tumour response in *BRAF* V600E mutated pLGGs than conventional therapies in some studies. To date, targeted therapy and precision medicine are promising avenues for paediatric gliomas with *BRAF* V600E and diffuse intrinsic pontine glioma with the H3K27M mutations. Given these shortcomings in the current treatments of paediatric gliomas, there is a dire need for novel therapies that yield a better therapeutic response. The present review discusses the diagnostic tools and the perspective of liquid biopsies in the detection of *BRAF* V600E and H3K27M mutations. An in-depth understanding of these biomarkers and the therapeutics associated with the respective challenges will bridge the gap between paediatric glioma patients and the development of effective therapies.

## 1. Introduction

Tumours of the central nervous system (CNS), derived from the brain and spinal cord, affect both adult and paediatric populations. From 2012 to 2016, the average annual age-adjusted incidence rate of CNS tumours (nonmalignant and malignant tumours) in the United States of America was 23.41 per 100,000 [1]. Gliomas that derived from glial cells constitute the majority of paediatric CNS tumours. According to the 2016 updated World Health Organisation (WHO) classification of CNS tumours, paediatric gliomas are classified into low-grade (grades I and II) and high-grade (grades III and IV) gliomas [2,3]. In this classification, new tumour entities are defined based upon molecular events and histological diagnosis. For instance, anaplastic astrocytomas and diffuse astrocytomas are further classified into isocitrate dehydrogenease (IDH)-mutant, IDH wild-type and not otherwise specific categories. Additionally, low-grade gliomas (LGGs) are more prevalent than high-grade gliomas (HGGs) in the paediatric population [4,5].

Paediatric low-grade gliomas (pLGGs) are slow-growing tumours and can be divided into primary glial tumours (astrocytoma and oligodendroglioma) and mixed glioneuronal tumours [4,5,6]. Astrocytomas, particularly pilocytic astrocytoma, are the most common type of pLGGs [5]. Pilocytic astrocytoma has been found in any location of the CNS, including the spinal cord, brainstem, cerebral hemisphere, optic chiasm, cerebellum and hypothalamus [5]. The genetic alterations involved in tumorigenesis and progression of pLGGs are different, and the prominent cause remains unknown. Various mechanisms, such as telomere maintenance, angiogenesis and the tumour microenvironment, as well as glioma-associated antigens, are still under investigation. Notably, pLGGs can be due to genetic mutations that upregulate the MAPK pathways [7,8,9]. This phenomenon was first observed in pilocytic astrocytoma patients with neurofibromatosis type I [8]. Since then, other genetic alterations, predominantly *BRAF-KIAA1549* fusion genes, which were involved in the MAPK pathway were identified [9]. *BRAF-KIAA1549* fusions are the most prevalent alterations in pLGGs, accounting for two-thirds of pLGGs, while *BRAF* V600E mutations only contribute to 17% of pLGGs [10,11,12,13]. pLGG patients with the *BRAF* V600E mutation respond poorly to both radiotherapy and chemotherapy, resulting in a lower progression-free survival (PFS) rate over five and 10 years in comparison to LGGs the of wild-type status [12,14].

Paediatric high-grade gliomas (pHGGs) are rare but highly aggressive, fast-growing and commonly associated with a dismal prognosis. The pHGGs are traditionally classified as anaplastic astrocytoma (grade III) and glioblastoma (GBM, grade IV) according to the old WHO classification [15]. Diffuse intrinsic pontine glioma, a brainstem glioma that mainly arises from pons, comprises diffuse astrocytoma, anaplastic astrocytoma and GBM, which are classified as grades II, III and IV gliomas, respectively [2,3]. pHGGs and diffuse intrinsic pontine glioma have similar histological appearances with poor prognosis; thus, they are always considered together, even though they are separate entities. The new 2016 WHO classification has included another grade IV glioma, a diffuse midline glioma with histone H3K27M mutation, including some diffuse intrinsic pontine gliomas [2,3]. Histone H3 mutations were first discovered in pHGGs in 2012, and the histone H3K27M mutation constitutes most of the somatic mutations in diffuse midline glioma [16]. Histone mutations are highly prevalent in pHGGs in comparison to other forms of malignancies [17,18]. The histone variants H3.3 and H3.1 are found in approximately 80% of diffuse intrinsic pontine glioma and other midline gliomas, with H3.2 being present to a lesser extent [19]. These histone mutations cause the substitution of lysine at position 27 with methionine (K27M) on the histone tail. The tumours with the *H3.3K27M* mutation are more aggressive clinically than those with the *H3.1K27M* mutation [19,20]. The K27M mutation causes tumour progression by interfering with the post-translational modifications of H3 [21,22,23]. A body of evidence has revealed that there are significant differences between pHGGs and adult HGGs. For instance, some adult HGGs are transformed from LGGs, but this situation is infrequent in pHGGs [5]. Besides, molecular alterations in children with pHGGs are commonly associated with histone H3 mutations, whereas IDH mutations, PTEN loss, and EGFR amplifications are commonly found in adult HGG but rarely happen in pHGGs [4,24].

To date, many molecular alterations in paediatric gliomas have been discovered, and they are potential biomarkers that hold great promise in the diagnosis, prognosis and predicting of better therapies for patients. Most importantly, understanding the pathogenesis of *BRAF* and histone H3 mutations and the feasibility of these biomarkers to be utilised as diagnostic and prognostic tools are more crucial, because these mutations are unique alterations that are distinct from adult gliomas and constitute the highest percentage in paediatric gliomas. Due to the anatomic location of diffuse intrinsic pontine glioma and other midline gliomas, surgical resection is unfeasible, with radiotherapy being the only current treatment that shows a temporary response in these tumours [25,26,27]. Given these shortcomings in the current treatment of paediatric gliomas, thus, there is an urgent need for the development of targeted therapies that focus on the tumours’ biological characteristics that would yield a better response in patients. Precision medicine is a promising avenue for paediatric gliomas, as they consist of fewer genetic drivers and are more genetically homogenous than adult gliomas; therefore, they have a higher potential for an efficacious response. This article reviews critically the use of *BRAF* V600E and H3K27M mutations as biomarkers and targeted options as well as their associated challenges in translating these biomarkers to clinical applications for paediatric gliomas. In addition, the diagnostic tools and the potential of liquid biopsy in the detection of *BRAF* V600E and H3K27M mutations for the diagnosis of paediatric gliomas are discussed intensively. The in-depth understanding of these biomarkers over the years and the therapeutics associated with the respective challenges discussed herein will bridge the gap between paediatric glioma patients and the development of effective therapies.

## 2. BRAF Mutations in Paediatric Low-Grade Gliomas

**Figure 1:**
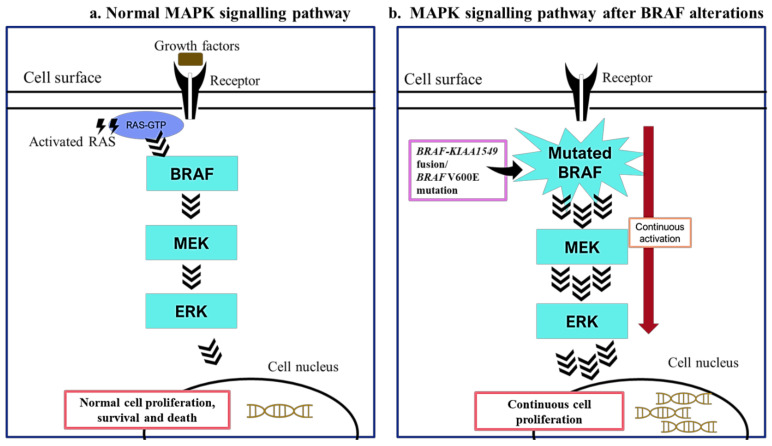
MAPK signalling pathway with and without *BRAF* alterations. In the normal MAPK signalling pathway (**a**) BRAF interacts with RAS, and the complex activates subsequent proteins in the pathway to promote normal cell proliferation. However, when the BRAF is mutated (**b**) it causes a continuous activation of MAPK pathway, which results in uncontrolled cell proliferation, leading to malignancies.

The BRAF protein that contains 766 residues is encoded by the *BRAF* gene located at 7q34 [28,29]. This protein transmits signals from cell surface receptors to cell nuclei and promotes cell growth. The BRAF protein is one of the key players in the MAPK signalling pathway, which regulates cell proliferation, differentiation and death (Figure 1) [30,31]. In a normal physiological process, BRAF is activated by interacting with RAS, which subsequently activates MEK 1 and MEK 2 that, in turn, activate extracellular regulated kinase 1 (ERK 1) and ERK 2 [32,33]. The activated ERK kinases then enter the nucleus to stimulate transcriptional pathways that promote cellular proliferation and survival. However, when the *BRAF* gene is mutated, the regulatory domain will be affected, and it remains in an activate configuration. As a result, this causes a continuous downstream activation of the MAPK signalling pathway, which promotes uncontrolled cell proliferation, eventually leading to tumourigenesis [34,35].

In pLGGs, the most common *BRAF* alterations are *BRAF-KIAA 1549* fusion and *BRAF* V600E mutation [36,37,38]. The *BRAF* V600E mutation is a point mutation, in which valine at position 600 is replaced with glutamic acid, whereas, for the *BRAF-KIAA1549* fusion, the *BRAF* gene fused with the *KIAA1549* gene, causing the removal of the regulatory domain [11,39]. The *BRAF* V600E mutation is a common mutation that occurs in many types of cancers, such as colorectal cancer, papillary thyroid cancer and melanoma [40]. Although this mutation has been reported in adult and paediatric gliomas, it is rarely found in adult gliomas. The *BRAF* V600E mutation has been detected in pilocytic astrocytoma, ganglioglioma, diffuse astrocytoma and pleomorphic xanthoastrocytoma [41]. Tumours with the *BRAF* V600E mutation can grow at any location in the CNS, and over 33% occur in the midline of the brain [12]. By contrast, the *BRAF-KIAA1549* fusion happens when the N-terminus and C-terminus of the proteins encoded by the *KIAA1549* and *BRAF* genes, respectively, are fused together [39]. The *BRAF-KIAA1549* fusions predominantly occur in pLGGs, and 70% are detected in pilocytic astrocytoma, which is usually located at the posterior fossa and cerebellum [5].

## 3. BRAF Mutations as Biomarkers in Paediatric Low-Grade Gliomas

*BRAF* alterations, especially V600E, have been studied intensively as potential diagnostic biomarkers for different types of cancers, including melanoma, colorectal cancer, papillary thyroid carcinoma and hairy cell leukaemia [40,42,43]. *BRAF* alterations are commonly found in pLGGs and are considered as valuable diagnostic markers. Tian et al. [39] established a quantitative RT-PCR for detecting the *BRAF-KIAA1549* fusion in 51 formalin-fixed paraffin-embedded (FFPE) tissue samples of pLGG. The sensitivity and specificity of the quantitative RT-PCR assay were 97% and 91%, respectively, as compared with fluorescence in situ hybridisation (FISH). Besides, the whole-genome sequencing of 39 pLGGs and low-grade glioneuronal tumours showed only two novel *BRAF* fusion genes: *BRAF-MACF1* and *FXR1-BRAF* [9]. Ryall et al. [44] employed the NanoString nCounter System for detecting duplications and different configurations of *BRAF-KIAA1549* fusions (97% sensitivity and 98% specificity) in 90 FFPE pLGG samples. In general, most of the diagnostic methods employed FFPE tissue samples, which might not provide sufficient nucleic acids. However, using a digital droplet PCR, the *BRAF-KIAA1549* fusion was detected from a minimal amount of FFPE tissue samples (one nanogram of DNA extracted) in pilocytic astrocytoma with a sensitivity and specificity of 100% compared with RNA sequencing [45]. Most recently, the *BRAF* V600E mutation in the FFPE samples of paediatric and adult LGGs were detected using a fully automated PCR with 100% sensitivity and specificity when compared with PCR and next-generation sequencing [46]. The application of liquid biopsies (blood, serum and cerebrospinal fluid (CSF)) has successfully bridged the gap between glioma patients and diagnosis with the identification of circulating tumour DNA [47,48]. Liquid biopsies of circulating tumour DNA in the plasma, serum and CSF isolated from 29 CNS paediatric patients revealed the presence of the *BRAF* V600E mutation [49].

Tumour prognostic biomarkers can be either nucleic acids, proteins, molecules or characteristics of the tumours, which allow the prediction of the clinical outcome of a patient without receiving any treatment. To date, the prognostic value of *BRAF* alterations, either *BRAF-KIAA1549* or *BRAF* V600E, in pLGGs remains questionable. Researchers have been arguing over the role of *BRAF* alterations in pLGGS. Two independent studies supported the role of *BRAF-KIAA1549* fusions as prognostic biomarkers [10,50]. The first studies by Hawkins et al. [10] involved a follow-up of 70 noncerebellar paediatric low-grade astrocytomas (diffuse astrocytoma, pilomyxoid astrocytomas and pilocytic astrocytoma) with less than three-quarters of complete resections beyond a year. Regardless of the patients’ ages, tumour locations and pathology, the results revealed that the clinical outcomes of patients with these fusions had a five-year PFS of 61%, significantly better than those without the fusions, which was only 18% [10]. Furthermore, Horbinski et al. [50] showed a significant improvement in PFS of all pLGGs harbouring *BRAF-KIAA1549* fusions as compared to those without the fusions. This may suggest that *BRAF-KIAA1549* fusions are the only factor that affects the clinical outcomes. Conversely, Colin and colleagues [51] argued that the prognostic factors, including patients’ ages, extent of tumour resections and tumour locations of the *BRAF-KIAA1549* fusions, are significantly associated with the clinical outcomes. A poorer clinical outcome occurred when the tumours were partially resected and located at hypothalamo-chiasmatic, as well as for infants or children younger than three years who were diagnosed with pilomyxoid astrocytomas harbouring these fusions.

A meta-analysis encompassing 1308 adults and paediatric gliomas from 11 studies revealed that children aged 0 to 16 years old with a *BRAF* V600E mutation have a favourable prognosis, with a hazard ratio of 0.51 [52]. However, age might be the main confounding factor in determining the clinical outcome of patients with *BRAF* V600E status. On the other hand, Mistry et al. [53] demonstrated that pLGGs harbouring *BRAF* mutations together with *CDKN2A* deletion have a high chance to transform into HGG, because *CDKN2A* deletion might alter the clinical behaviour of the *BRAF* V600E mutant tumours. A further study performed by Lassaletta et al. [12] revealed that the five-year PFS of pLGG patients with a *BRAF* V600E mutation and *CDKN2A* deletion was low (approximately 24%), while those without the deletion had a five-year PFS of 68.7%. Additionally, the 10-year PFS of *BRAF* V600E patients with *CDKN2A* deletion and without the deletion were approximately 0% and 46%, respectively [12]. The authors claimed that *BRAF* V600E mutations with other associated alterations might influence the prognosis.

According to the aforementioned studies, collectively, the role of *BRAF* alterations as a prognostic biomarker for pLGGs is still debatable. There are still many factors that have yet to be investigated thoroughly, including confounding factors, the relationship of *BRAF* mutations and other associated genetic alterations, as well as *BRAF* alterations as an independent prognostic indicator in affecting the clinical outcomes of patients.

## 4. Histone H3 Mutations in Paediatric High-Grade Gliomas

**Figure 2:**
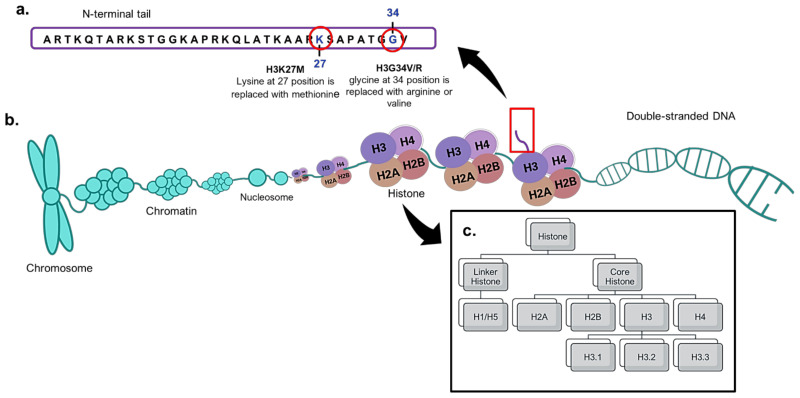
Structure of histone and histone H3 mutations. (**a**) Histone H3 mutations (H3K27M and H3G34V/R) at the N-terminus of H3. (**b**) Histones pack human DNA to form nucleosomes and chromatins. (**c**) Types of histones and variants.

Histones are essential proteins that pack DNA molecules into nucleosomes and further condense these primary packaging units into chromatins [54,55]. These essential proteins are divided into core histones (H2A, H2B, H3 and H4) and linker histones (H1 or H5) [55]. The core histones are found at the centre of nucleosomes, while linker histones interact with the nucleosomes and the linker DNA located between two nucleosomes. The centre of the nucleosomes contains an octamer of the four core histones in dimeric forms (Figure 2). Among the four core histones, mutations of H3 were first detected in pHGGs in 2012 [16]. H3 has three variants or isoforms, namely H3.1, H3.2 and H3.3, which are encoded by different genes. In pHGGs, mutations commonly take place at the N-terminal end of H3. This site plays a role in post-translational modifications such as methylation, phosphorylation, ubiquitination, acetylation, the regulation of DNA replication, DNA repair and RNA transcription [55]. The most common histone H3 mutation involves the substitution of lysine at position 27 with methionine (H3K27M) in variants H3.1 and H3.3, encoded by the *HIST1H3B* and *H3F3A* genes, respectively [56]. In addition, other mutations also take place in histone H3.3, of which glycine 34 is replaced with valine or arginine (H3.3G34V/R), mainly involving the *H3F3A* gene [57]. In general, the prevalence of H3K27M in pHGGs is higher than H3.3G34V/R. The H3.3 and H3.1 mutations were detected in FFPE tissues from 143 paediatric high-grade astrocytomas using immunohistochemistry, and this result was in good agreement with that determined with the pyrosequencing method [58].

## 5. H3K27M Mutations as Biomarkers in Paediatric High-Grade Gliomas

The missense H3K27M mutation is mostly found in pHGGs derived from midline locations of the brain, including the brainstem, thalamus, pons, cerebellum and spinal cord [57]. A point mutation in the histone H3 variant (H3.3 or H3.1) is commonly found in diffuse intrinsic pontine glioma [16,17,20,59,60]. Wu et al. [16] reported that 78% of H3K27M mutations are detected in diffuse intrinsic pontine glioma, approximately 60% and 18% of which involve the *H3F3A* gene and *HIST1H3B* gene, respectively. Most recently, Dufour et al. [61] studied 49 patients with paediatric diffuse midline glioma and discovered that 80% of the tumour carried H3K27M mutations, of which 63.6% and 15.9% of the *H3F3A* and *HIST1H3B* genes, respectively, were mutated. The studies demonstrated that *H3F3A* and *HIST1H3B* mutations are common in the pHGGs derived from midline locations of the brain. These mutations are hardly found in adult malignant gliomas, thus making H3K27M a distinct biomarker for paediatric gliomas.

As compared to a brain biopsy, the locations for the sampling of liquid biopsies are less invasive and easily accessible, which saves time and costs. Recently, liquid biopsies are used to identify the H3K37M mutation in pHGGs (Table 1). An in vitro study to isolate circulating tumour DNA from cancerous DIPG007 cells cocultured with normal human astrocytes was performed by Stallard et al. [62]. The circulating tumour DNA released into the culture media was detected with a droplet digital PCR (ddPCR) using specific probes against the *H3F3A*K27M mutation [62]. In another study using CSF-derived DNA from paediatric midline gliomas, H3 mutations were detected using Sanger sequencing and nested PCR targeting *H3F3A*, with a sensitivity and specificity of 87.5% and 100%, respectively [63]. Furthermore, Pan et al. [64] isolated the circulating tumour DNA from the CSF of brainstem gliomas (*n* = 57, of which 23 samples were diffuse midline glioma), which led to the detection of multiple tumour-specific mutations, including *H3F3A, TP53, ATRX, PDGFRA, HIST1H3B, FAT1, PPM1D, ACVR1, NF1, IDH1* and *PIK3CA.* With respect to the sensitivity of mutation detection, CSF-circulating tumour DNA gave rise to a higher sensitivity (100%) as compared to plasma-circulating tumour DNA (38%) [64]. Most importantly, 82.5% of the 57 CSF-circulating tumour DNA samples harboured a single tumour-specific mutation, indicating the potential of CSF-circulating tumour DNA in detecting mutations in brainstem gliomas. This finding was corroborated with another study that detected 88% of the CSF, and plasma-circulating tumour DNA harboured H3K27M mutations in diffuse midline glioma patients [65]. However, the amount of circulating tumour DNA in plasma was lower than that of the CSF. This could be due to the blood–brain barrier (BBB) that restricts circulating tumour DNA from entering the blood and the dilution of circulating tumour DNA by the lysis of white blood cells during storage before analysis [66].

Paediatric groups that suffer from diffuse intrinsic pontine glioma containing H3K27M mutations have a worse prognosis with a median survival of 0.73 years, as compared to those harbouring wild-type tumours: 4.59 years [67]. Similarly, children with diffuse midline glioma also show poor prognosis, with a median overall survival (OS) of 0.78 years [61]. Generally, many studies concluded that pHGGs with H3K27M mutations are always associated with a worse prognosis. However, Castel et al. [67] demonstrated that molecular alterations and clinical behaviour within *H3.1K27M* and *H3.3K27M* could affect the patients’ prognoses. Most recently, a retrospective study introduced three prognostic markers to refine diffuse midline glioma prognosis: the loss of *17p*, *PDGFRA* amplification and a complex chromosomal profile [61]. These three markers are associated with worse survival in diffuse midline glioma patients. Karremann et al. [20] studied 85 paediatric DMG patients whose tumours were distributed in the pons, thalamus and spinal cord. The researchers demonstrated that H3K27M mutations were associated with worsen clinical outcome regardless of anatomic location, the extent of surgical resection and histopathological grading. This indicates that H3K27M mutations in paediatric diffuse midline glioma patients can be used as important prognostic biomarkers.

## 6. Perspective of Liquid Biopsy

Traditionally, clinical symptoms and MRI are mainly used to diagnose paediatric gliomas. However, these diagnostic methods cannot provide information on chromosomal mutations and rearrangements of the tumours, which is crucial in diagnosing pHGGs associated with H3 mutations. Tissue biopsies for conventional and molecular diagnoses pose a huge challenge for surgeons due to the anatomical location of pHGG in the midline of the brain. Besides, the risks of brain biopsy include infection, haemorrhage and death. Even though a brain biopsy can be successfully performed, the amount of tissue samples may not be enough for histopathology and molecular diagnosis. Hence, a liquid biopsy is an alternative method that can be used to obtain the vital information of tumours from body fluids such as blood (serum or plasma), CSF and urine while being a less noninvasive approach [68]. The body fluids contain biomaterials such as cell-free DNA, circulating tumour cells, circulating tumour DNA, fragmented peptides and microRNAs that are released from the tumours.

Although the popularity of liquid biopsies as a source of biomarkers is increasing, many studies only used a limited number of samples. The validity and potentiality of liquid biopsies thus require a large cohort or multicentre cohort studies. So far, from the abovementioned studies, CSF is one of the primary sources of biomarkers due to a higher concentration of circulating tumour DNA, and lumbar puncture is the mandatory procedure in getting CSF samples. However, this procedure is quite invasive for children with gliomas, of which those present in the brain will increase the intracranial pressure, with a risk of developing brain herniation, as the withdrawal of CSF may create an acute pressure gradient. Even though this complication is rare, as a safety precaution, medical professionals do not usually perform this procedure for routine screening. Hence, the number of CSF samples available is relatively limited.

To date, many established diagnostic methods are used for detecting H3 mutations from different sources of biopsy samples. However, the whole-genome sequencing of liquid biopsies is gaining popularity for detecting H3 mutations in pHGGs owing to the precision of the method to determine single and multiple mutations in cancerous cells, as well as the accessibility of tumour materials in liquid biopsies. Bettegowda and colleagues [69] demonstrated that patients with aggressive tumours or metastatic cancers have a higher concentration of circulating tumour DNA in plasma as compared to low-grade cancers. If this phenomenon is true for paediatric cancers, it could explain why liquid biopsies are not popular in pLGGs, as the circulating tumour DNA concentration in plasma could be too low for detecting *BRAF* V600E mutations. However, to rule out this assumption, studies have to be performed to determine the concentration of circulating tumour DNA in pLGGs and pHGGs. On the other hand, although liquid biopsy is gaining popularity as a source of tumour material in gliomas with encouraging data [68], it cannot wholly replace FFPE samples, because its tumour components are only a subset of FFPE samples. Therefore, more studies are still required to validate the accuracy of biomarker detections in liquid biopsies. Collectively, not many diagnostic and sampling methods have been developed for detecting the *BRAF* V600E mutation, as compared to *BRAF-KIAA1549* mutations. Hence, the development of diagnostic assays for detecting the *BRAF* V600E mutation in pLGGs using liquid biopsies is the way forward.

## 7. Therapy in Paediatric Gliomas

### BRAF Inhibitors in pLGGs with BRAF V600E

Treatment strategies for pLGGs should focus on minimising both short- and long-term morbidity. The optimal management of pLGGs is influenced by the locations, sizes and modes of presentation of the tumours, in addition to their histological subtypes. Generally, surgical resection is the first line of therapy for well-circumscribed lesions in the cerebral or cerebellar cortex that are sizeable and superficial. According to a prospective multi-institutional study in patients with pLGGs, the five-year PFS is more than 90% in children that underwent gross total resection, in comparison to the disease progression in approximately 50% of those with less extensive resections [70]. Furthermore, the location of the tumour has a strong impact on its outcome. Patients with superficial tumours in the cerebral and cerebellar cortices are more manageable by gross total resection without significant morbidity, resulting in a better prognosis. In contrast, gross total resection is too risky to function in patients with tumours that are deeper in the basal ganglia, optic pathways, diencephalon or brainstem. Thus, only a biopsy or partial resection is possible in most cases. Adjuvant therapies are often required for these deep, unresectable lesions to prevent tumour progression [6,70]. The current adjuvant therapies are mostly limited to radiotherapy and chemotherapy, with a three-year PFS of about 70%. However, both treatments are associated with significant morbidity due to their high toxicities [71,72,73,74,75]. Radiotherapy is effective for the majority of pLGGs, but it is associated with side effects such as cognitive deficits, endocrinopathies and secondary malignancies. Therefore, it is usually only advocated when other treatment options have been exhausted, particularly in children under the age of ten [6,76,77]. Chemotherapy using carboplatin and vincristine is currently one of the most established regimens among pLGG patients [6]. However, in a large clinical and genetic institutional study, the use of standard chemotherapy in pLGG patients with the *BRAF* V600E mutation recorded a 10-year PFS of 27% (95% confidence interval (CI), 12.1–41.9%) in comparison to 60.2% (95% CI, 53.3–67.1%) in pLGG patients of wild-type status (hazard ratio, 2.04; 95% CI, 1.45–2.88; *p* < 0.001) [12]. In addition to the various toxicities associated with chemotherapy, including immunosuppression, it is also a logistically inconvenient form of treatment due to its weekly schedule of intravenous therapy [6].

The use of *BRAF* V600E inhibitors in six pLGG patients with the *BRAF* V600E mutation who experienced tumour progression after being treated with conventional therapy demonstrated a significant cytoreduction of 49% to 80% and prolonged survival in comparison to a 25% tumour reduction after chemotherapy [12]. These patients were treated with *BRAF* V600E inhibitors with a median follow-up of 18.5 months. Subsequently, one of the six patients in the study had notable tumour progression upon discontinuing the *BRAF* inhibitor. However, the same patient reported a rapid clinical and radiological response upon restarting the drug [12].

Dabrafenib is a selective *BRAF* V600E inhibitor with a significant clinical response in a variety of *BRAF* V600E adult malignancies, including melanoma, non-small cell lung cancer and anaplastic thyroid cancer [78,79,80]. The drug is approved for the treatment of unresectable or metastatic *BRAF* V600E melanoma as a single agent or in combination with the MEK inhibitor Trametinib [80,81]. The positive clinical response to Dabrafenib in these BRAF V600E adult malignancies has opened doors to several trials of the drug in paediatric gliomas [71], in which the initial findings demonstrated that Dabrafenib in *BRAF* V600^-^relapsed or refractory pLGGs showed an overall response rate of 41% [82]. In a phase I/IIa open-label study (NCT01677741), the use of Dabrafenib in *BRAF* V600E pLGGs with recurrence, progression or resistance post-chemotherapy (*n* = 32, age 1–18 years old) showed an overall response rate of 44% (95% CI, 26–62%) among all patients [71]. The treatment demonstrated a one-year progression-free survival rate of 85% (95% CI, 64–94%), portraying the clinical efficacy of the drug in this subgroup of patients. However, the patients reported common side effects, including fatigue, dry skin, pyrexia, maculopapular rash, migraine, hypotension, back pain, arthralgia, thrombocytopenia, lymphocytopenia, neutropenia, increased serum alkaline phosphatase and weight gain. However, these adverse effects were well-controlled by the reduction or interruption of the drug dosage and supportive care. This paediatric patient group did not report any cases of squamous cell skin carcinoma, keratoacanthoma or treatment-related deaths, as reported in adult patients. This observation may suggest that Dabrafenib has acceptable drug tolerability within paediatric patients.

Vemurafenib is a small molecule that acts as a competitive and selective inhibitor of the ATP-binding domain of the *BRAF* V600E mutation in cells [83]. The drug has been approved by the Food and Drug Administration (FDA) for treating metastatic melanomas with the same mutation. Vemurafenib (720 mg for 28 days, as an off-label drug) was first tested on a 12-year-old Caucasian boy with a right frontoparietal GBM (*BRAF* V600E) treated with conventional modalities, who developed tumour recurrence 2.5 years from diagnosis [84]. Serial MRI brain monitoring indicated a complete tumour regression after four months of treatment, and the effect was sustained for another six months. Due to the high prevalence of the *BRAF* V600E mutation in paediatric gliomas, this case study provided a positive indication for Vemurafenib in the treatment of these tumours. In a study, 57% of seven pLGG patients (four patients with gangliomas, one patient each with xanthoastrocytoma, ganglio-neurocytoma and pilocytic astrocytoma; median age diagnosis of 75.2 months) with the BRAF V600E mutation responded to Vemurafenib, which was well-tolerated, with dermatological toxicity as the main adverse effect [83]. Notably, all the responding patients demonstrated an improvement in neurological function after two weeks of treatment with Vemurafenib.

## 8. HDAC Therapy in pHGGs and Diffuse Intrinsic Pontine Glioma

Unlike adult HGGs, there is no clear establishment of standard treatment protocols in pHGGs. Gross total resection of the tumour is done when possible, which generally improves survival in pHGGs patients who receive adjuvant radiotherapy (except in infants and very young children due to the possibility of neurodevelopmental toxicity) after resection. However, surgical resection is not feasible with diffuse intrinsic pontine glioma and midline HGGs due to their location and infiltrative nature [85]. Fractionated external beam radiotherapy (doses from 54 to 60 Gy) is the standard treatment for midline gliomas with the H3K27 mutation [85]. To date, chemotherapy has not portrayed added benefits in treating diffuse intrinsic pontine glioma. The role of the chemotherapeutic agent, Temozolomide remains unclear in pHGGs with the H3K27 mutation, as these gliomas are O-6-methylguanine-DNA methyltransferase (MGMT) unmethylated. Hence, it is likely to be futile in treating them [85,86,87]. Numerous clinical trials that aimed at improving the prognosis of diffuse intrinsic pontine gliomas over several decades have failed to improve the OS (median of 9–11 months) and survival rate between two to three years (<10%) of the disease [85,87].

Histone deacetylases (HDACs) can regulate the histone acetylation that is responsible for gene expression by inducing changes in chromatin conformation [20,88]. HDACs also regulate other non-histone substrates such as oncogenes and tumour-suppressor proteins. Therefore, the deregulation or mutations of HDAC may promote human malignancy [20]. Owing to these facts, HDAC inhibitors may be used to prevent tumour progression. Panobinostat is a class I, II and IV pan-HDAC inhibitor. It has portrayed superior inhibitory activity in vivo to FDA-approved HDAC inhibitors such as vorinostat and romidepsin [89]. In 2014, Panobinostat was approved by the FDA for the treatment of multiple myeloma [20]. Several clinical trials have used the drug for various malignancies, including thyroid cancer, prostate cancer, renal cancer, colorectal cancer, non-small cell lung cancer, neuroendocrine tumours, lymphoma and leukaemia [20]. The drug is also being tested in a clinical trial (NCT01324635) for adult CNS malignancies in combination with stereotactic radiation therapy [20]. Recently, it has portrayed preclinical efficacy and has entered a phase I clinical trial (NCT02717455) for recurrent or progressive paediatric diffuse intrinsic pontine glioma [20]. In the preclinical study, among the 83 drugs screened on 16 patient-derived paediatric diffuse intrinsic pontine glioma cultures, Panobinostat was among the most promising agents [20,90]. The drug temporarily inhibited the growth of a H3.3K27M tumour at a dosage of 10 mg/kg, three times a week, to a Nonobese diabetic/severe combined immunodeficiency (NOD-SCID) patient-derived orthotopic xenograft diffuse intrinsic pontine glioma mice model. However, the overall survival of these mice was not reported. Furthermore, the finite number of drugs used in this study and the failure of in vitro systems to model the cellular complexity of the tumour microenvironment that invariably affected the drug efficacy are some limitations of this study. However, the data in the study provided a promising avenue of treatment for a presently untreatable disease.

Once considered a dream drug, ONC201 is a molecule that induces the tumour necrosis factor (TNF)-related apoptosis-inducing ligand (TRAIL), which is specific for killing tumour cells, but does not harm non-neoplastic cells [91,92]. The molecule acts as a selective antagonist for dopamine receptor D2/3, which is capable of triggering p53-independent cell death in preclinical models of HGGs [85,93,94]. Its ability to antagonise DRD2 receptors lead to the activation of an integrated stress response coupled with inactivation of the Akt/ERK pathway, thus inhibiting tumour progression [85,92,95,96]. The first patient who received the drug in a phase II clinical trial (NCT02525692) was a 10-year-old girl with a diffuse intrinsic pontine glioma with the H3K27M mutation. She had a decreased tumour volume by 26%, 40% and 44%, respectively, on MRIs taken every eight weeks [85]. Additionally, the patient’s ipsilateral hearing was reported to be restored, and the patient’s facial palsy improved from House-Brackmann Grade IV to Grade I at 16 weeks. Even though the patient eventually passed away after almost two years of treatment with ONC201 due to new lesions in the thalamus and cerebellum and progression of the primary tumour, this led to the initiation of an expanded access programme of the drug conducted by Chi et al. [85]. Among the four paediatric patients with H3K27M gliomas in the study who were treated with adjuvant ONC201 after radiation therapy, two diffuse intrinsic pontine glioma patients remained progression-free for a minimum of 53 and 81 weeks, respectively. However, some of the caveats in the study include reporting single-patient experiences from a heterogeneous cohort of patients with limited time for a follow-up. In addition, the effects of other treatments postradiotherapy and prior to tumour recurrence in some patients may confound the results of the study due to the possibility of pseudoprogression. Nevertheless, the clinical outcomes and radiographic responses from this study and the drug’s ability to target the H3K27 mutation specifically provide an initial or potential clinical proof-of-concept and a rationale for further clinical testing of the agent in the treatment of these gliomas [85].

The chemically modified enhanced derivatives of ONC201, namely ONC206 and ONC212, are shown to be more efficacious by enhancing OTX016 (a BRBD4 antagonist) in suppressing in vitro and in vivo glioblastoma via the serine-one carbon-glycine pathway and transcription factor ATF4 [95]. This observation may prompt the beneficial use of imipridone derivatives in pLGGs and pHGGs in combination with other drug molecules. Similar to Panobinostat, ONC201, an imipridone derivative, has given high expectations of therapeutic efficacy in human patients. However, most of these studies using Panobinostat, ONC201 and its derivatives are either at a preclinical stage or are used on a case study basis. The ambiguity in the safe and tolerable dosage of these drugs in treating patients continues to remain a fundamental issue. Furthermore, the reported case studies and ongoing clinical trials are yet to provide concrete evidence of the therapeutic relevance and safety of these drugs. Hence, it is warranted to be used in future clinical studies, such as large cohort and multicentre studies, either alone or in combination with other therapeutic drugs.

## 9. Challenges in Paediatric Gliomas

Even though almost all the above-mentioned drugs have shown efficacy in vivo, their clinical use may be significantly hampered by their ability to reach the intended site of action and tumour heterogeneity. Gliomas are frequently located in unresectable parts of the brain. Additionally, the BBB physiologically protects the brain tissue from being exposed to substances in the circulation and, thus, may impede the gliomas’ exposure to drugs [6,97]. Having low molecular weight, low protein binding and high lipophilicity will make a drug more likely to cross the BBB with good bioavailability in response to the tumours. Drugs that are not substrates of efflux proteins in the BBB are more likely to be sustained in the brain tissue than if otherwise [97]. Recent advances in technology, including genetic sequencing; the delivery of treatment and imaging such as nanodiamond systems, targeted radiotracers, small drug molecules targeting protumour pathways and microfluid devices for single-cell processing have enabled more accuracy and accessibility in precision medicine. However, there is a considerable gap between these new technologies and their applications in paediatric patients; thus, innovative ways to bridge these differences are vital in the advancement of effective therapies for paediatric malignancies. Glioma animal models will assist in identifying molecular pathways involved in tumourigenesis and may subsequently support the potential treatment strategies. However, these in vivo studies often fail due to the different pharmacokinetic profiles between the animals used and humans. The animal models normally do not completely reflect human biological properties and they lack tumour heterogeneity compared to those found in humans [97,98]. Furthermore, the lack of paediatric tumour materials and intratumoural heterogeneity may confound the accurate diagnosis of certain tumours.

The complex genetic features of glioma tumours and their microenvironments often cause resistance to both conventional and novel therapies. This, together with the rarity of paediatric CNS malignancies in comparison to other malignancies, discourage pharmaceutical companies to invest in research for new drug therapies. Based on the above-mentioned studies, there is sufficient justification for the integration of targeted therapy in the treatment of paediatric gliomas. However, paediatric gliomas are rarer than other malignancies, which pose a huge challenge in attracting adequate funding to conduct further clinical trials on novel drugs. Hence, there are a lack of research studies and clinical trials in this area.

Moreover, as reported in the case studies above, most of these drugs were initially used as off-label drugs in these patients, with evidence of drug efficacy obtained from studies targeting treatments in adult tumours originating elsewhere in the body, even though they share the same mutations of interest as the paediatric gliomas. Hence, unfavourable outcomes of the drugs may be attributed to the use of the drugs in a different target group than the primary study. The response of BRAF inhibitors may be limited in some patients due to drug resistance [35]. Even as BRAF inhibitors suppress BRAF monomers in *BRAF* V600E malignant cells, they may also cause RAF dimerisation in non-BRAF-mutated cells [99] and the cells containing splice variants of the mutation [100]. Increased RAF dimerisation leads to paradoxical activation of the MAPK pathway. This can induce BRAF inhibitor resistance, which may be overcome by the concurrent use of MEK inhibitors [43,101]. The use of BRAF/MEK inhibitor combination therapy in treating paediatric malignancies with the *BRAF* V600E mutation is minimal, and several clinical trials are still underway. Data on the combination treatment are limited to case reports. However, to date, there are no published reports of the combination therapy used as an upfront treatment for paediatric malignancies. The efficacy and safety of Dabrafenib/Trametinib combination therapy was studied for *BRA*F V600E-mutated LGGs and relapsed/refractory HGG paediatric patients [71,102,103]. A case report on a 16-year-old adolescent female with a recurrent anaplastic ganglioma positive for the *BRAF* V600E mutation showed a significant response to 150-mg Dabrafenib (orally, twice daily) and 2-mg Trametinib (orally, four times daily) after being intolerant to Vemurafenib [104]. The patient developed a diffuse morbilliform rash after one week of treatment with 960 mg (orally, twice daily) of Vemurafenib and started on a BRAF/MEK inhibitors combination after six months due to an interval increase in the size of the lesion. Moreover, the malignant lesion completely resolved after eight weeks of the combination therapy, with a subsequent MRI done at six months with minimal adverse effects to the combination treatment as compared to Vemurafenib monotherapy. The results of the study suggest that the combination therapy of Dabrafenib/Trametinib has a better safety profile and efficacy than Vemurafenib monotherapy. Thus, it is a promising treatment strategy that is worth being further investigated in the treatment of *BRAF* V600E-mutated paediatric gliomas.

## 10. Conclusions and Future Perspectives

Over the past decade, many molecular characteristics and alterations of paediatric gliomas have emerged rapidly. Currently, animal models are commonly used in conducting in vivo studies for paediatric gliomas. However, these studies usually fail due to pharmacokinetic and biological differences in the animals used and the paediatric patients. Hence, it is imperative to use models that represent paediatric patients accurately to ensure the clinical efficacy of the novel drugs in treating paediatric gliomas. It is of the utmost importance that the drugs of choice can cross the BBB in sufficient amounts to inhibit tumour progression. Therefore, future trials should consider both targeted patient selection, as well as BBB penetration of the drugs, in order to assess their clinical efficacy in paediatric patients successfully.

pHGGs with histone H3 mutations are always associated with worse clinical outcomes, while the prognostic values of *BRAF* mutations remain questionable due to many contradictory findings. There is a rapid development of precision medicine in the diagnosis and treatment of paediatric gliomas. Several clinical trials for targeted therapies aiming at specific mutations in paediatric gliomas are underway, and the future treatments for these tumours look promising. Due to the low number of participants available for the clinical research of paediatric malignancies, the accurate preclinical development of models is vital in the discovery of novel therapeutic options. The current classification of brain tumours is mainly based on microscopic morphology and immunohistochemistry. The genomic classification of these tumours will certainly improve the sensitivity and specificity of the diagnostic assays and, thus, will provide insights into accurate targeted therapies for these genetic subtypes.

The in-depth understandings of the molecular mechanisms coupled with various molecular methods (FISH, real-time PCR, NanoString nCounter system and whole-genomic sequencing) that provide high sensitivity and specificity have led to the discoveries of *BRAF* and histone H3 mutations as biomarkers in paediatric gliomas. In order to function as a useful diagnostic biomarker, sampling procedures must also be taken into consideration, in addition to these biomarkers being easily and rapidly detectable by the current molecular diagnostic methods. So far, liquid biopsies are considered less invasive than tissue biopsies in obtaining samples for biomarker detection in the paediatric population. As of now, studying the prognostic value of specific mutations alone is certainly inadequate. Therefore, investigations on these mutations should be performed together with other coexisting alterations to gain a better understanding of the relationship of their underlying pathogenesis and prognosis. The search for irrefutable diagnostic and prognostic biomarkers in liquid biopsies for paediatric gliomas should be of great interest to many neuroscientists, neuropathologists and oncologists in the coming decade.

## Figures and Tables

**Table 1:** A summary of the liquid biopsies used for detecting H3 mutations in paediatric high-grade gliomas (pHGGs). CSF: cerebrospinal fluid.

Paediatric Gliomas	Biomaterial	Biosources	Diagnostic Methods	Genetic Mutations and Findings	References
Paediatric brain tumour (*n* = 11), including diffuse midline gliomas (*n* = 6)	Circulating tumour DNA and genomic tumour DNA	CSF	Sanger sequencing and nested PCR	*H3F3A*K27M mutations were detected in four out of six samples using the newly established methods.	[63]
Diffuse intrinsic pontine glioma	Circulating tumour DNA	CSF	Droplet digital PCR	*H3F3A*K27M mutations were detected.	[62]
Brainstem gliomas (*n* = 57) and diffuse intrinsic pontine glioma (*n* = 23)	Circulating tumour DNA	CSF	Deep sequencing	Multiple tumour-specific mutations such as *H3F3A, TP53, ATRX, PDGFRA, HIST1H3B, FAT1, PPM1D, ACVR1, NF1, IDH1* and *PIK3CA* were detected. H3F3A was the highest mutated gene, with a percentage of approximately 48%. Approximately 9% of altered *HIST1H3B* genes were detected.	[64]
Diffuse midline glioma (*n* = 48)	Circulating tumour DNA	79 plasma samples and 30 CSF samples	Droplet digital PCR	*BRAF, PPM1D, H3F3A, HIST1H3B, ACVR1* and *PIK3R1* mutations were detected. Eighty-eight percent of the CFS and plasma circulating tumour DNA(ctDNA) harboured H3K27M mutations. The amount of ctDNA in plasma was lower than that of the CSF.	[65]

## Data Availability

Not applicable.

## Abbreviations

BBB	blood–brain barrier
CI	confidence interval
CSF	cerebrospinal fluid
ERK	extracellular regulated kinase
FISH	fluorescence in situ hybridisation
HDAC	histone deacetylase
IDH	isocitrate dehydrogenase
MAPK	mitogen-activated protein kinase
MGMT	O-6-methylguanine-DNA methyltransferase
pLGGs	paediatric low-grade gliomas
BRAF	B-Raf Proto-Oncogene or V-Raf Murine Sarcoma Viral Oncogene Homolog B
CNS	central nervous system
EGFR	epidermal growth factor receptor
FFPE	formalin-fixed paraffin-embedded
GBM	glioblastoma
HGGs	high-grade gliomas
LGGs	low-grade gliomas
MEK	mitogen-activated protein kinase kinase
pHGGs	paediatric high-grade gliomas
PTEN	phosphatase and tensin homologue
